# Laparoscopic surgery for duodenal perforation due to a diverticulum with heterotopic pancreas: a case report

**DOI:** 10.1186/s40792-022-01460-3

**Published:** 2022-06-01

**Authors:** Shu Tsukihara, Shinji Onda, Kyonsu Son, Daisuke Ito, Hironori Kanno, Toshiaki Morikawa, Nobuyoshi Hanyu, Ken Eto

**Affiliations:** 1 Department of Surgery, Tokyo General Hospital, 3-15-2 Ekoda, Nakano-ku, Tokyo, 165-8906 Japan; 2 Department of Surgery, The Jikei University School of Medicine, 3-25-8 Nishi-Shimbashi, Minato-ku, Tokyo, 105-8461 Japan

**Keywords:** Heterotopic pancreas, Duodenal perforation, Laparoscopy

## Abstract

**Background:**

Heterotopic pancreas (HP) refers to the presence of abnormally located pancreatic tissue without any anatomic or vascular continuity with the main body of the pancreas. HP can occur in the gastrointestinal tract and be complicated by gastrointestinal bleeding, pancreatitis, obstruction, or malignant generation. Specifically, perforation of the gastrointestinal tract because of HP is extremely rare.

**Case presentation:**

A 91-year-old woman was diagnosed with duodenal perforation, and an emergency laparoscopic operation was performed. The operative findings indicated a tumor and duodenal wall perforation. The tumor and the perforated site were resected with a linear stapler. Histopathological examination revealed the presence of HP tissue in the submucosal layer around the diverticulum without any signs of inflammation. The perforated site was not covered by HP tissues, and the duodenal wall might have been weaker than the other areas, which could have caused the internal pressure to increase and led to the perforation.

**Conclusions:**

Preoperative HP diagnosis is difficult, and it is crucial to consider HP as the differential diagnosis in gastrointestinal perforations. The duodenal diverticula can be perforated due to increased internal pressure of the duodenum caused by the imbalanced localization of HP.

## Background

Heterotopic pancreas (HP) and aberrant, ectopic, or accessory pancreas refer to the presence of pancreatic tissues outside of their normal location and without anatomic or vascular continuity with the pancreas itself [1]. HP is an uncommon condition and is rarely diagnosed because it is generally asymptomatic. This disorder is frequent in the abdominal region (30% in the duodenum, 25% in the stomach, 15% in the jejunum, 3% in the ileum, and 6% in the Meckel diverticulum) [2]. At times, HP causes gastrointestinal bleeding, pancreatitis, obstruction, or malignant generation [3]. HP mostly occurs as a submucosal lesion, but it is also seen sporadically in the muscularis propria and subserosa [4]. The von Heinrich classification defines three types of HP, as follows: the ectopic pancreas can be composed of ducts, acini, and Langerhans islands (Heinrich’s type I), duct and acini without islands (Heinrich’s type II), or only several ducts, some of them cystically dilated, without exocrine or endocrine components (Heinrich’s type III heterotopy) [5].

Two cases are known so far, including the present one describing a duodenal perforation caused by HP [6]. In patients with perforated peptic ulcers who are stable, a laparoscopic approach is recommended by the World Society of Emergency Surgery guidelines [7]. This study presents a case of laparoscopically treated safe duodenal perforation caused by a diverticulum with HP.

## Case presentation

**Fig. 1:**
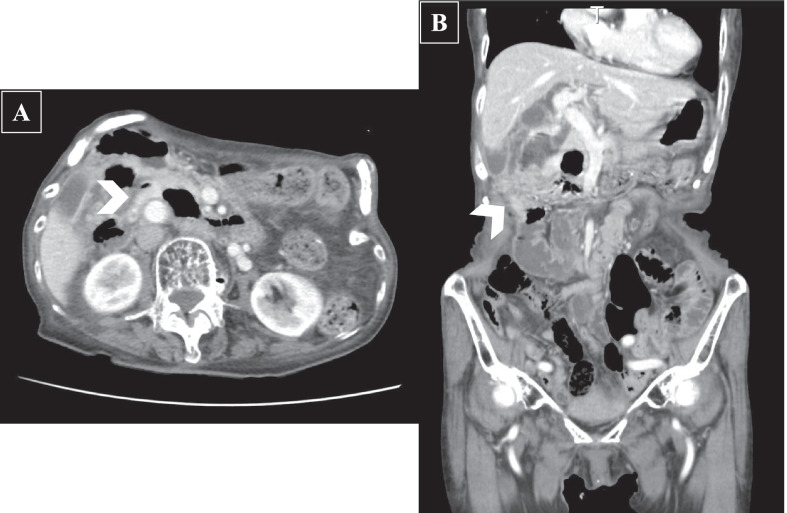
Contrast material-enhanced computed tomography. **A** Axial contrast-enhanced CT shows enhancement of thickened duodenal wall (*arrowhead*). **B** Reformatted coronal contrast-enhanced CT image also detects the thickened duodenal wall with inflammation involving periduodenal fat (*arrowhead*)

**Fig. 2:**
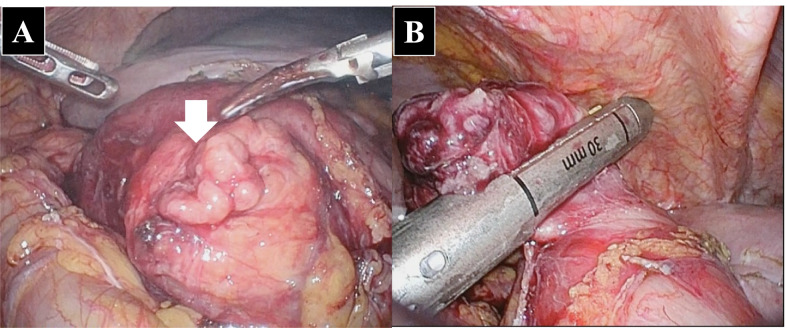
Intraoperative findings. **A** Intraoperative findings: the perforated anterior duodenal wall (*white arrow*) was detected, and intestinal fluid was observed to be flowing out. **B** The perforated site was resected using an autosuture device

**Fig. 3:**
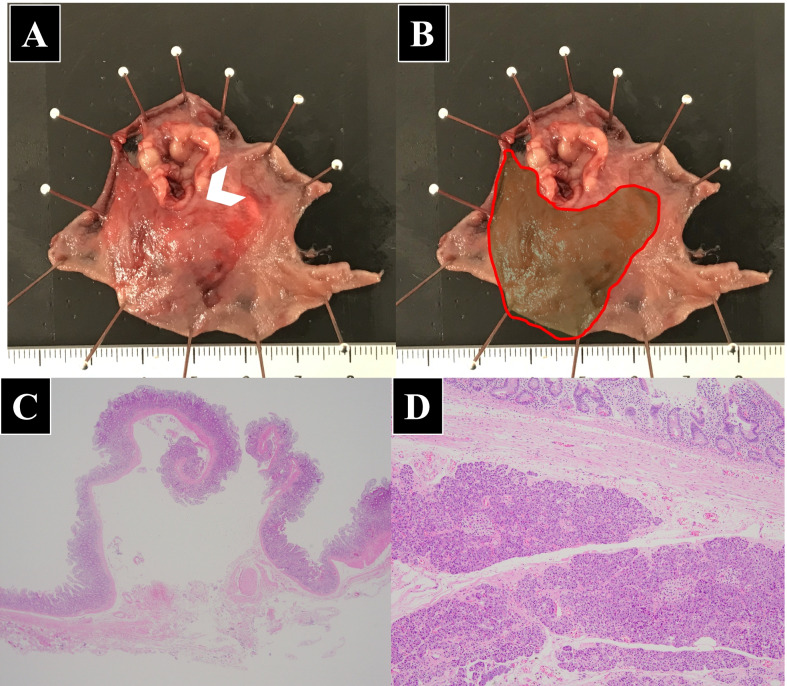
The resected specimen. **A** The resected specimen contained the perforated site (*arrowhead*). **B** The area marked with the *red line* was covered by HP in the submucosa. The surgical margin was positive for HP. **C** The microscopic view of the specimen indicated that the perforated mucosa was not covered by HP (hematoxylin and eosin ×50). **D** Additionally, it revealed the presence of normal, well-recognized pancreatic tissue, including islets of Langerhans, in the surrounding areas (hematoxylin and eosin ×100)

A 91-year-old woman was transferred to the hospital with complaints of right upper abdominal pain. The patient had a history of constipation and osteoporosis treated with magnesium oxide (990 mg/day) and eldecalcitol (0.75 μg/day). Moreover, she had a history of early gastric cancer, which was treated with endoscopic mucosal resection 9 years ago. On physical examination, she had tenderness and distension in the upper abdomen along with rebound tenderness and muscle guarding. The biochemical parameters were within normal limits, except for leukocytosis (white blood cells, 12.9 × 10^3^/μL) and C-reactive protein elevation (5.7 mg/dL). Abdominal computed tomography indicated ascites, enhancement of the duodenal wall, and free air around the duodenum (Fig. 1). Emergency endoscopy was performed, which revealed a perforation in the second portion of the duodenum. Thus, an emergency laparoscopic surgery was performed. The operative findings showed the presence of a perforated duodenal wall wrapped by the omentum. The wrapped omentum was dissected, and a tumor was identified in the perforated duodenal wall. The tumor and the perforated site were resected with a linear stapler (Fig. 2). The surgical duration and intraoperative blood loss were 98 min and 15 mL, respectively. Postoperatively, the patient developed mild acute pancreatitis, but recovered well with conservative treatment. Consequently, she was discharged on postoperative day 15. The surgical specimen showed a perforated duodenal diverticulum, and histopathological examination suggested the presence of HP tissue in the submucosal layer around the diverticulum without any signs of inflammation. The specimen comprised pancreatic acinar cells, ducts, and islets of Langerhans (Fig. 3). A pathological diagnosis of Heinrich type I HP was made. The patient has been free from recurrence 15 months after the operation.

## Discussion

HP is a rare condition and is frequently detected incidentally during surgery or histopathological examination. HP is often asymptomatic, and the clinical symptoms are not specific. Symptoms related to HP are divided into two types: a combination of mass effect and local and systemic complications due to activated pancreatic enzymes [8]. The former, which causes bowel or biliary obstructions, is attributed to the presence of the tumor. The latter, which causes pancreatitis or pancreatic neoplasm, is because of the pancreatic tissues. Currently, two reports of duodenal perforation related to HP, including the present case, two reports of gastric perforation, and five reports of jejunal perforation caused by HP are available [6, 9–15]. Gunjaca et al. documented the first case of duodenal perforation due to HP with acute and chronic pancreatitis [6]. The two cases of gastric perforation were caused by peptic ulcers in the stomach [9, 10]. On the contrary, the jejunal perforation reported by Shiratori et al. occurred in the diverticula along with severe inflammation [15]. HP has been reported as a cause for intestinal perforation not only in adults, but also in a neonate with multiple congenital anomalies [12]. The details of the previous cases and ours are summarized in Table 1.

**Table 1:** Summary of the nine cases of gastrointestinal perforations related to HP

Author (year)	Age/sex	Perforation site	Heinrich’s classification	Surgical procedure	Probable cause of perforation
Petersen et al. (2005)	86/F	Jejunum	I	Open partial resection	Acute inflammation
Pal et al. (2008)	0/F	Jejunum	II	Open partial resection	Ulceration
Gurocak et al. (2009)	19/F	Stomach (antrum)	II	Open distal gastrectomy	Ulceration
Gunjaca et al. (2010)	51/M	Duodenum	ND	Open distal gastrectomy	Chronic inflammation
Wong et al. (2011)	62/M	Jejunum	ND	Open partial resection	Inflammation and bacterial infection
Hamabe et al. (2012)	67/M	Jejunum	I	Open partial resection	Necrotic change of the tumor containing HP
Fukino et al. (2012)	68/F	Stomach (antrum)	I	Open distal gastrectomy	Ulceration
Shiratori et al. (2017)	83/F	Jejunum	I	Open partial resection	Diverticulum with inflammation
Current case (2021)	91/F	Duodenum (second portion)	I	Laparoscopic partial resection	Diverticulum without inflammation

*ND* not described

The perforated site in the diverticulum in the present case was surrounded by HP tissues in the submucosal layer. Most duodenal diverticula are extraluminal and acquired rather than congenital [16]. Taking the patient’s age and the pathological findings into consideration, the diverticulum also was extraluminal. Since HP is a congenital anomaly [17], the HP had been present for a long time before the perforated diverticulum arose. Unfortunately, the diverticulum was generated in the HP area, resulting in the imbalanced localization of HP. The imbalanced HP localization in the duodenum might have led to the perforation. The perforated site was not covered by HP tissues, and the duodenal wall might have been weaker than the other areas, which could have caused the internal pressure to increase and led to the perforation. Betzler et al. indicated the necessity for surgery in symptomatic HP cases because of the possibilities for acute and chronic pancreatitis, the occurrence of pseudocystic changes, or even a malignant transformation to adenocarcinoma or acinar cell carcinoma [18]. Duodenal perforations can also occur in people with conditions such as duodenal diverticula, duodenal ischemia, infectious diseases, and autoimmune conditions, including Crohn’s disease, scleroderma, and vasculitis (e.g., abdominal polyarteritis nodosa) [19]. Preoperatively, a diagnosis of duodenal perforation caused by the diverticulum was made based on upper endoscopic findings. Finally, histopathological examination revealed that the resected specimen was the duodenum, including HP. Though HP with inflammation was detected in many cases [6, 11, 13, 15], the HP had no inflammation in this case. As mentioned above, HP causes symptoms in two ways: the presence of the tumor or the pancreatic enzymes. In this case, it is possible that the presence of the tumor is associated with perforation by generating the imbalance of the intraluminal pressure.

Although abdominal computed tomography showed enhancement of the duodenal wall (Fig. 1), which was not specific for HP. Endoscopic ultrasonography seems to be useful for diagnosis of HP [17]. However, it is true that it could be performed in limited institutions in an emergency case. Intraoperative endoscopy could have provided a chance for detecting the presence of HP. The patient suffered from pancreatitis after the operation. Considering that the surgical margin was positive in histopathological examination, there is a possibility that the HP remained. The residual HP might have been damaged and released pancreatic enzymes, causing the pancreatitis. In this case, pathological diagnosis during surgery would have confirmed the safety of this operation; however, it is often difficult during emergency operations. Because pancreatoduodenectomy in emergency and elderly patients had some risks, it was not performed.

In terms of the surgical procedure, omentum implantation to treat perforated sites is common in upper gastric perforation. However, in this case, the patient’s omentum was not sufficiently rich for an implantation. The laparoscopic procedure seemed to be feasible because the technique was similar to the ways of repairing duodenal perforations resulting from other causes, such as ulcerations or diverticula.

## Conclusion

An extremely rare case of duodenal perforation related to HP is reported. Since preoperative HP diagnosis is difficult, it is crucial to consider HP as a differential diagnosis in case of gastrointestinal perforations. The duodenal diverticula can be perforated due to increased internal pressure of the duodenum caused by the imbalanced localization of HP.

## Data Availability

Not applicable.

## Abbreviation

HP: Heterotopic pancreas
